# Supplementary material for: γ-TuRC asymmetry induces local protofilament mismatch at the RanGTP-stimulated microtubule minus end
Source: EMBO J. 2024 Apr 10;43(10):7. doi: 10.1038/s44318-024-00087-4 (PMC11099078; doi:10.1038/s44318-024-00087-4)
Supplement: Supplementary file 1 — Table EV1 [file 44318_2024_87_MOESM1_ESM.docx]

**Table EV1 - Cross-correlation of the atomic models of open, partially closed and closed γ-TuRCs indicates that particle supplementation does not bias reconstruction of the MT-capping γ-TuRC**.

To judge the influence of particle supplementation on the cryo-EM density of the MT minus end, we systematically compared a range of reconstructions obtained with different refinement strategies with atomic models of open, partially closed and closed γ-TuRCs by cross correlation. The best correlating model for each reconstruction is marked in bold.

A Before any particle supplementation, the reconstruction of the γ-TuRC at the MT minus end correlates best with the model of the partially closed γ-TuRC.

B-D The final reconstruction of the γ-TuRC at the MT minus end shows highest correlation towards the partially closed atomic model, irrespective of whether the refinement was supplemented with particle images of the open γ-TuRC (B), simulated particle images of the hypothetical closed γ-TuRC (C) or a combination of supplemented particles (D).

E We performed a local refinement step after (B), retaining the supplemented open particles, in which refinement was only focused on spokes 1-8, i.e., where the open and partially closed atomic models are highly similar (Appendix Fig. S4). This leads to a reconstruction of the MT-capping γ-TuRC that correlates best with the partially closed γ-TuRC.

F Refinement of only MT-capping γ-TuRC particles with global sampling following (B) yields a reconstruction that best correlates with the partially closed γ-TuRC.

G, H As controls, the reconstruction of only supplemented open particles shows highest cross-correlation towards the atomic model of the open γ-TuRC (G), and similarly, the reconstruction of only supplemented closed particles shows highest cross-correlation towards the hypothetical atomic model of the closed γ-TuRC (H). Given the difference in cross-correlation between open and closed atomic models in these controls, the observed differences in cross-correlations in (A-F) are in the expected range for the partially closed γ-TuRC as an intermediate conformation between open and closed.

| **γ-TuRC conformation** | Open | Partially closed | Closed (hypothetical) |
| --- | --- | --- | --- |
| **Reconstruction** |  |  |  |
| A) Reconstruction of γ-TuRC before supplementation of particles | 0.67 | **0.70** | 0.67 |
| B) Final γ-TuRC-focused reconstruction after supplementation of open particles | 0.82 | **0.85** | 0.78 |
| C) Final γ-TuRC-focused reconstruction after supplementation of simulated closed particles | 0.80 | **0.84** | 0.83 |
| D) Final γ-TuRC-focused reconstruction after supplementation of 50% open particles and 50% simulated closed particles | 0.83 | **0.86** | 0.83 |
| E) Reconstruction of γ-TuRC after supplementation of open particles, followed by local refinement focused on spoke 1-8 | 0.82 | **0.84** | 0.79 |
| F) Reconstruction of γ-TuRC after refinement with supplementation of open particles (B), followed by refinement with global sampling without particle supplementation | 0.75 | **0.78** | 0.74 |
| G) Reconstruction of only supplemented open particles | **0.89** | 0.85 | 0.79 |
| H) Reconstruction of only supplemented simulated closed particles | 0.75 | 0.75 | **0.81** |
