# Supplementary material for: γ-TuRC asymmetry induces local protofilament mismatch at the RanGTP-stimulated microtubule minus end
Source: EMBO J. 2024 Apr 10;43(10):7. doi: 10.1038/s44318-024-00087-4 (PMC11099078; doi:10.1038/s44318-024-00087-4)
Supplement: Supplementary file 2 — Appendix [file 44318_2024_87_MOESM2_ESM.pdf]

Appendix

Table of contents

Appendix Figure S1 - Purification of γ-TuRC-capped MT minus ends from Xenopus laevis egg extract.	2
Appendix Figure S2 - RanGTP treatment drastically increases the number of γ-TuRC-capped MTs isolated from X. laevis egg extract.	3
Appendix Figure S3 - Cryo-EM reconstruction of the MT lattice distant from the capped minus end.	4
Appendix Figure S4 - Detailed structural analysis of the γ-TuRC at the MT minus end.	5
Appendix Figure S5 - Extended analysis of the minus end of capped MTs.	6
Appendix Figure S6 - Cryo-EM processing workflow for γ-TuRC-capped MTs not subjected to the shortening procedure.	7
Appendix Figure S7 - Binding of CAMSAP2 to the lattice and γ-TuRC-capped minus ends of MTs nucleated through the RanGTP pathway in X. laevis egg extract.	8
Appendix Figure S8 - Binding of CAMSAP2 to the lattice and γ-TuRC-capped minus ends of MTs nucleated in vitro.	10
Appendix Table S1 - Mass spectrometry analysis of γ-TuRC-capped MTs isolated from X. laevis egg extracts.	12
Appendix Table S2 - Cross-correlation of the atomic models of open, partially closed and closed γ-TuRCs indicates that particle supplementation does not bias reconstruction of the MT-capping γ-TuRC, Paclitaxel-stabilised particles only.	13
Appendix Table S3 - Cross-correlation of the atomic models of open, partially closed and closed γ-TuRCs indicates that particle supplementation does not bias reconstruction of the MT-capping γ-TuRC, DTX-stabilised particles only.	14
Appendix Table S4 - Cross-correlation of the atomic models of open, partially closed and closed γ-TuRCs indicates that particle supplementation does not bias reconstruction of the MT-capping γ-TuRC, particles that were not subjected to the shortening procedure only.	15
References	16

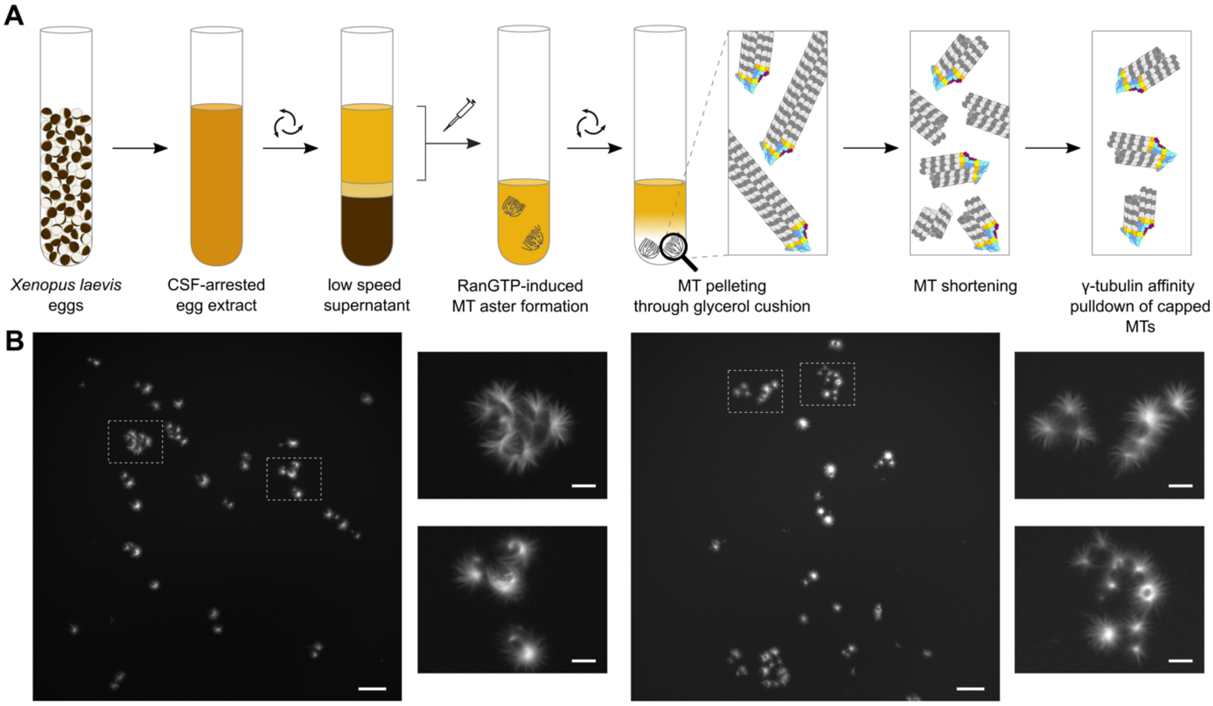

Appendix Figure S1 - Purification of γ-TuRC-capped MT minus ends from *Xenopus laevis* egg extract.

A Schematic representation of the workflow for purification of γ-TuRC-capped MTs from *Xenopus laevis* egg extract.

B Sample fluorescence microscopy images used for judging aster-forming capacity of *Xenopus laevis* egg extracts, a prerequisite for further purification of γ-TuRC-capped MT minus ends. Scale bars represent 100 µm in full images and 25 µm in zoomed cut-outs.

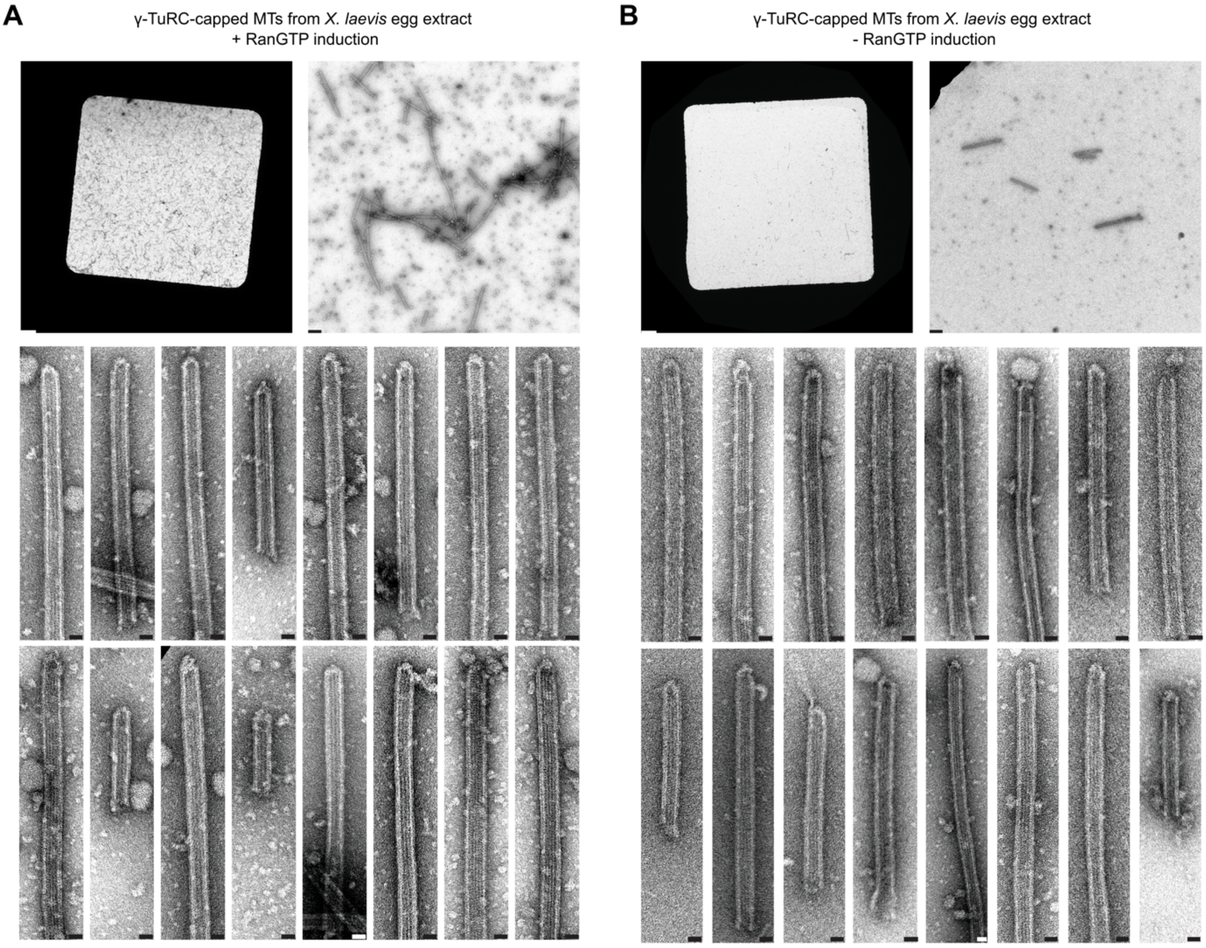

Appendix Figure S2 - RanGTP treatment drastically increases the number of γ-TuRC-capped MTs isolated from *X. laevis* egg extract.

A,B Representative negative stain EM micrograph at the grid square level (top left, scale bars 3 µm), at intermediate magnification (top right, scale bar 200 nm) and a gallery of cut-outs at high magnification (bottom, scale bar 20 nm) of γ-TuRC-capped MTs purified with (A) or without (B) RanGTP induction. MTs were stabilised with Paclitaxel.

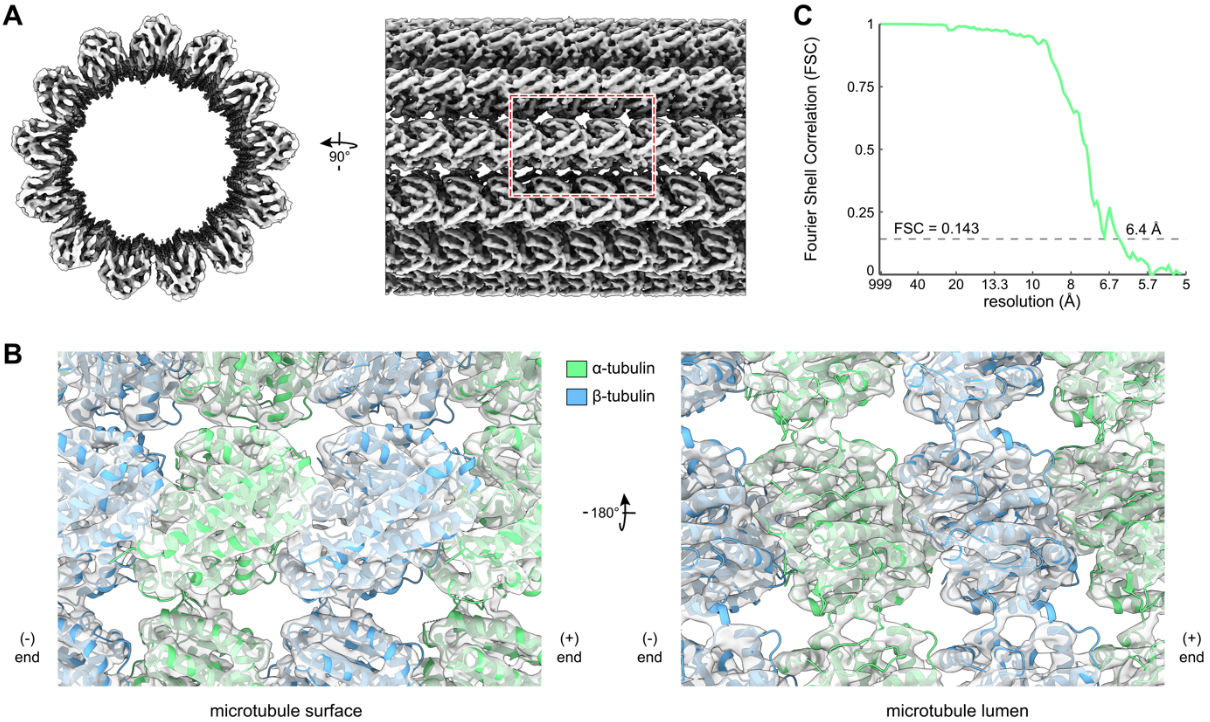

Appendix Figure S3 - Cryo-EM reconstruction of the MT lattice distant from the capped minus end.

A Overview of the MT lattice density of Paclitaxel-stabilised MTs distant from the capped minus end, clearly resolving defined secondary structure elements, indicating the MT lattice is structurally intact. Dashed box indicates the area focused on in panel (B).

B Dimer-wise fit of an atomic model of mammalian α- and β-tubulin (PDB 6EW0 (Manka & Moores, 2018)) into the density shown in (A), viewed from the MT surface (left) and lumen (right).

C Fourier Shell Correlation (FSC) curve used for determining the resolution (as specified in the plot) of the reconstruction in panel (A) at threshold FSC=0.143 (indicated by a dashed line).

Source data is available for this figure.

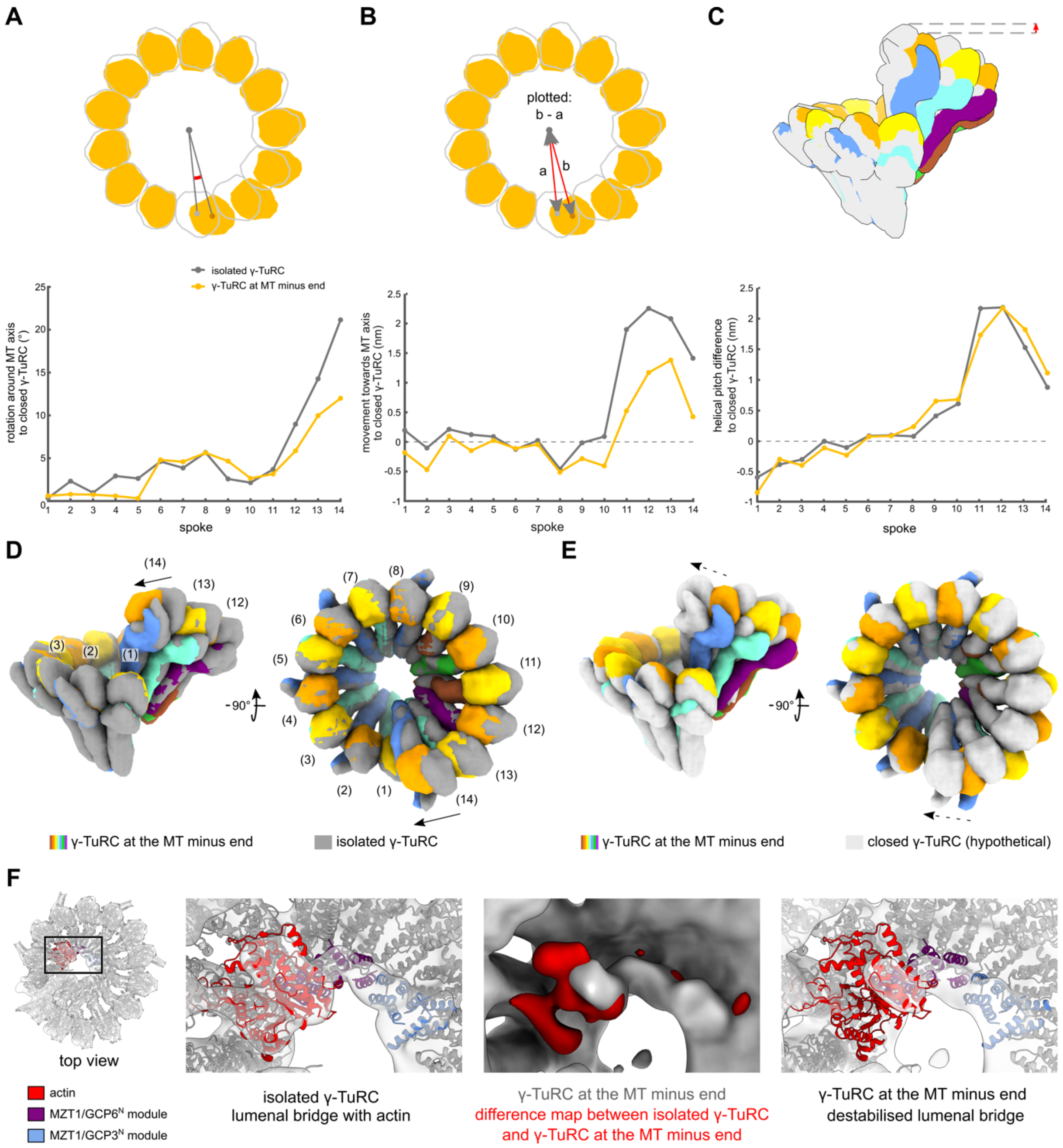

Appendix Figure S4 - Detailed structural analysis of the γ-TuRC at the MT minus end.

A-C Rotation around the MT axis (A), translation towards the MT axis (B) and downward change in helical pitch (C) required to convert the isolated γ-TuRC (dark grey) or the γ-TuRC at the MT minus end (orange) to the hypothetical, fully closed γ-TuRC for each spoke. Inset schematics represent the quantified parameters. Parameters measured from the center of mass of γ-tubulin.

D,E Comparison of the γ-TuRC at the MT minus end with the isolated γ-TuRC (PDB 6TF9(Liu *et al*, 2020), (D)) and the hypothetical fully closed γ-TuRC (E), generated from EMD 2799 (Kollman *et al*, 2015) (see Methods). Lumenal bridge components are omitted. Molecular surfaces generated from atomic models using the molmap function in UCSF ChimeraX (Goddard *et al*, 2018).

F Zoom-in of the lumenal bridge in the reconstruction of the isolated γ-TuRC (left panel) and the γ-TuRC at the MT minus end (right), both superposed with the atomic model of the isolated *X. laevis* γ-TuRC (PDB 6TF9) (Liu *et al.*, 2020). Difference density (red, middle panel) superposed with the reconstruction of the γ-TuRC at the MT minus end (grey) highlights that the MZT1/GCP6^{N} and MZT1/GCP3^{N} modules are well resolved, while density for actin is significantly reduced. All reconstructions were low-pass filtered to 17 Å. Colouring as indicated; components outside the lumenal bridge are shown in grey.

Source data is available for this figure.

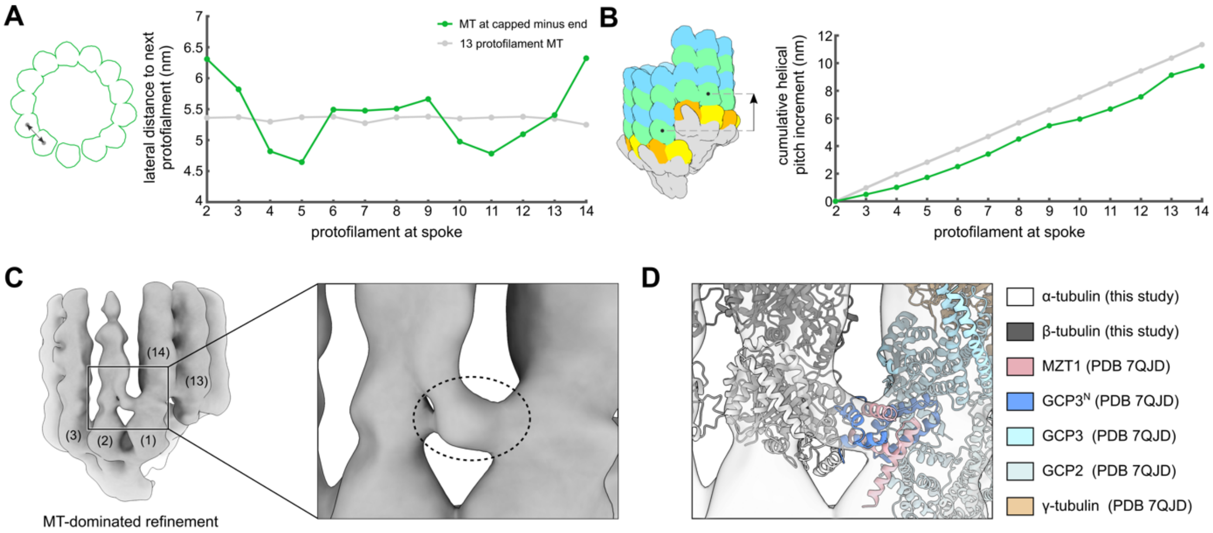

Appendix Figure S5 - Extended analysis of the minus end of capped MTs.

A Quantification of the lateral distance between the first α-tubulin in the protofilament at spoke N to the first α-tubulin in the protofilament at spoke N+1.

B Quantification of the helical pitch increment for the protofilament at spoke N compared to the protofilament at spoke 2. Colouring as in panel (A). All parameters were calculated using the center of mass of the α-tubulin molecule closest to the γ-TuRC, with schematics illustrating the plotted parameter.

C Zoom-in on the density connecting the protofilament at spoke 2 with spoke 1 and 14 (highlighted by dashed circle) in the MT-dominated reconstruction of the γ-TuRC-capped MT. Spoke numbering is indicated.

D The model of the γ-TuRC-capped MT generated in this study (white and dark grey) as well as spokes 13 and 14 with the terminal MZT1/GCP3^N module of the human recombinant γ-TuRC (PDB 7QJD (Wurtz *et al*, 2022)) superposed to the reconstruction shown in panel (C). Spokes 13, 14 and the MZT1/GCP3^N module were docked as a rigid body. Colouring scheme indicated.

Source data is available for this figure.

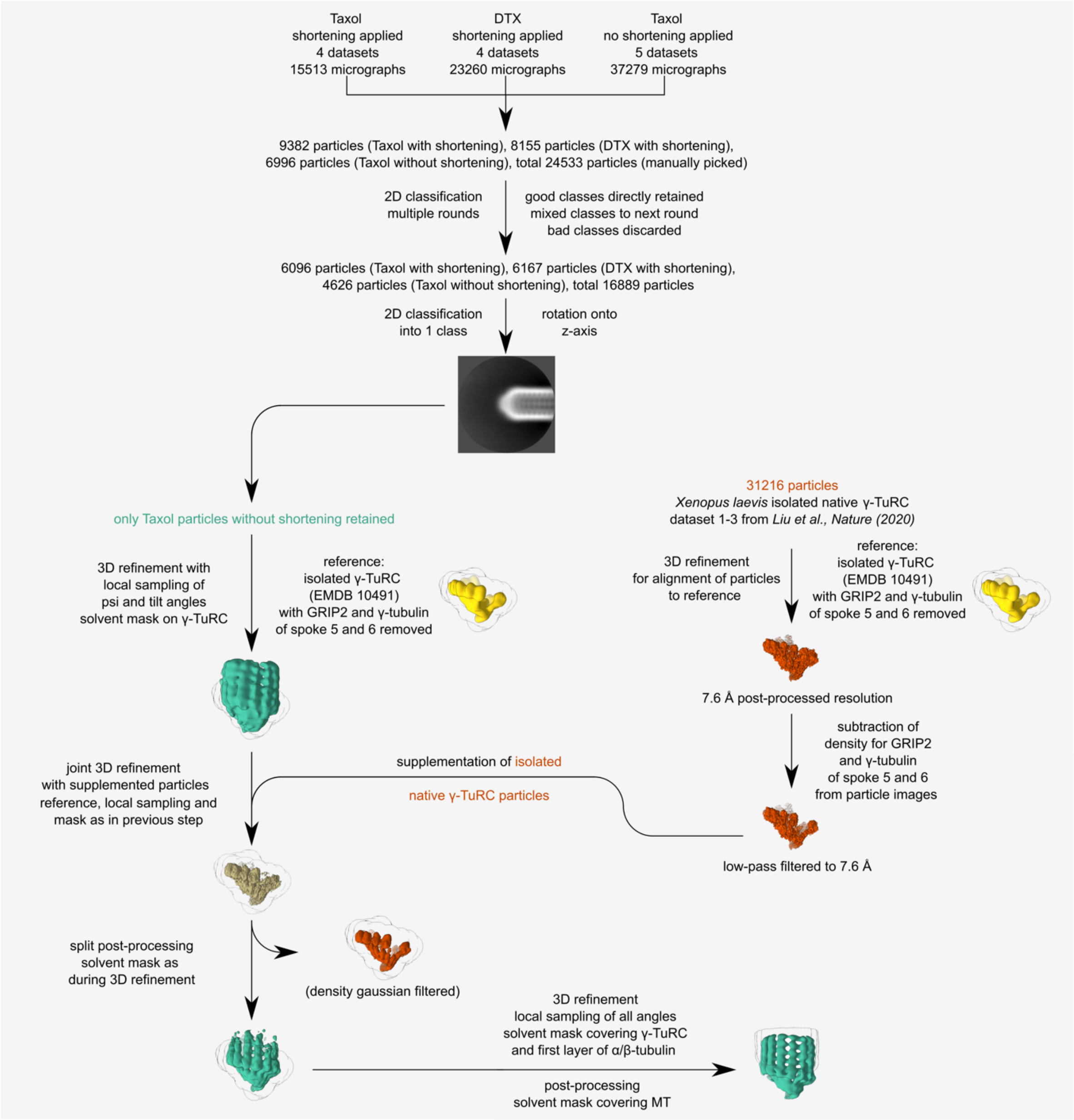

Appendix Figure S6 - Cryo-EM processing workflow for γ-TuRC-capped MTs not subjected to the shortening procedure.

Detailed image processing scheme. Colours of text and reconstructions indicate the particles used for reconstruction; capped MT minus ends in green, isolated γ-TuRCs in red, a mixture of both in khaki and reference densities in yellow. Densities are shown for schematic purposes.

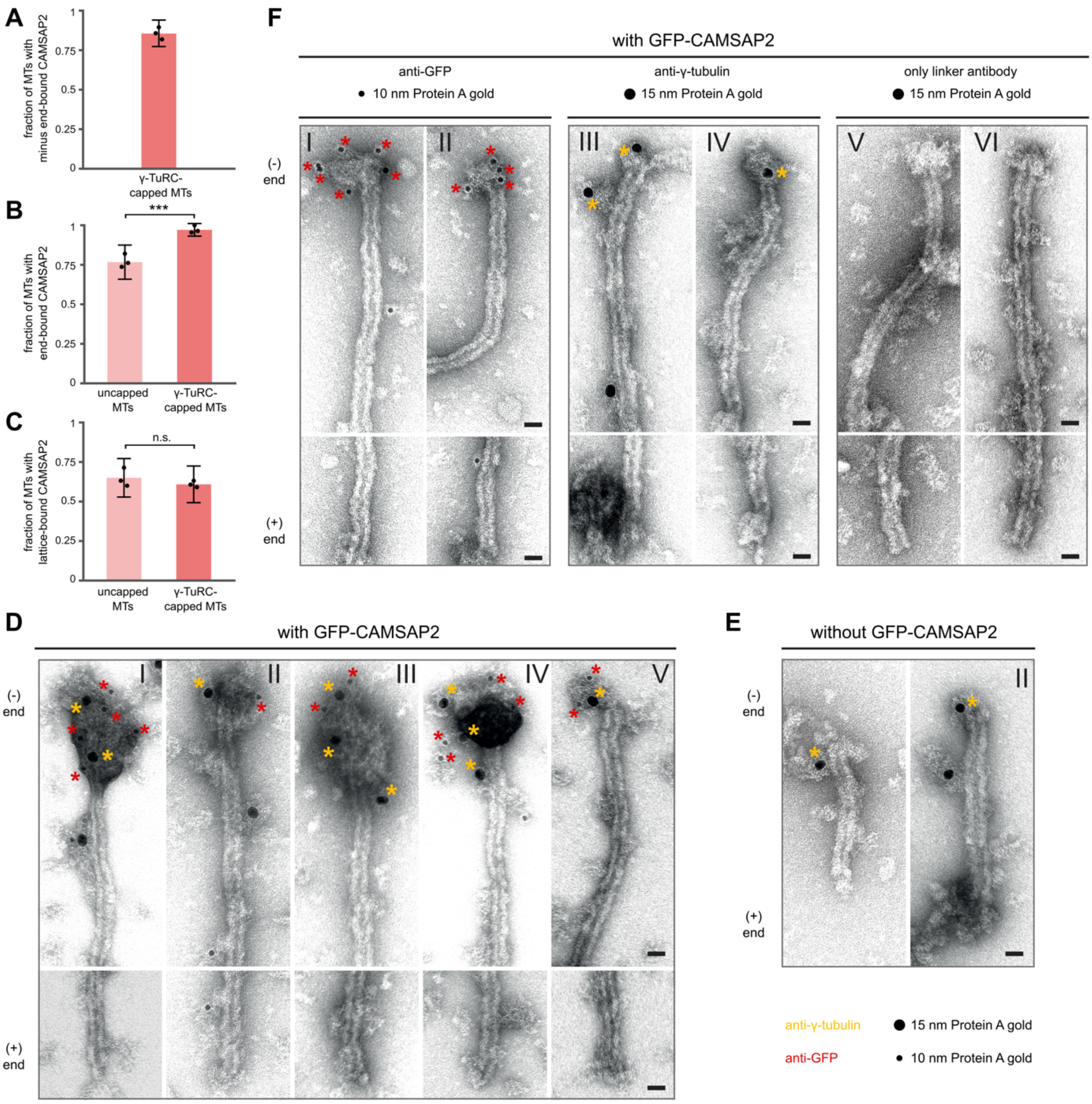

Appendix Figure S7 - Binding of CAMSAP2 to the lattice and γ-TuRC-capped minus ends of MTs nucleated through the RanGTP pathway in *X. laevis* egg extract.

A Fraction of γ-TuRC-capped MTs with CAMSAP2 binding to the minus end observed by multi-colour fluorescence microscopy (within 1 µm of the MT end; n=69, three biological replicates). Example fluorescence microscopy images are shown in Figure 4A.

B Fraction of γ-TuRC-capped MTs and uncapped MTs with CAMSAP2 binding to either end observed by multi-colour fluorescence microscopy (within 1 µm of the MT end; n=69 for capped MTs, n=60 for uncapped MTs, p=0.000418, three biological replicates).

C Fraction of γ-TuRC-capped MTs and uncapped MTs with CAMSAP2 binding to the MT lattice observed by multi-colour fluorescence microscopy (i.e., >1 µm from either MT end; n=69 for capped MTs, n=60 for uncapped MTs, p=0.3181). Dots in (A), (B) and (C) indicate mean values of the three biological replicates; data are shown as mean with 95% confidence interval for all individual data points. *** p<0.001, n.s. non-significant. Significance determined using a one-tailed Welch's t-test.

D, E Immunogold labelling of γ-TuRC-capped MTs isolated from *X. laevis* egg extract incubated with GFP-CAMSAP2. GFP-CAMSAP2 and γ-tubulin both localise to the same end of γ-TuRC-capped MTs, which is not observed in samples where GFP-CAMSAP2 is absent (E). Red (CAMSAP2) and yellow (γ-TuRC) asterisks indicate gold beads at the MT minus end. Additional density at MT ends may be attributed to staining of antibodies as well as potential accumulation of condensates of CAMSAP2 (Imasaki *et al*, 2022).

F γ-TuRC-capped MT minus ends isolated from *X. laevis* egg extract were incubated with GFP-CAMSAP2 and labelled with single gold conjugate: anti-GFP antibody labelled with 10 nm Protein A gold conjugated to rabbit anti-goat antibody (left), anti-γ-tubulin antibody labelled with rabbit anti-mouse antibody that was subsequently labelled with 15 nm Protein A gold (middle) and labelling of only rabbit anti-mouse antibody with 15 nm Protein A gold (right). Red (CAMSAP2) and yellow (γ-TuRC) asterisks indicate gold beads at the MT minus end. Dots in legend indicate approximate observed size of respective gold beads. Scale bar in (D), (E) and (F) represents 25 nm.
Source data is available for this figure.

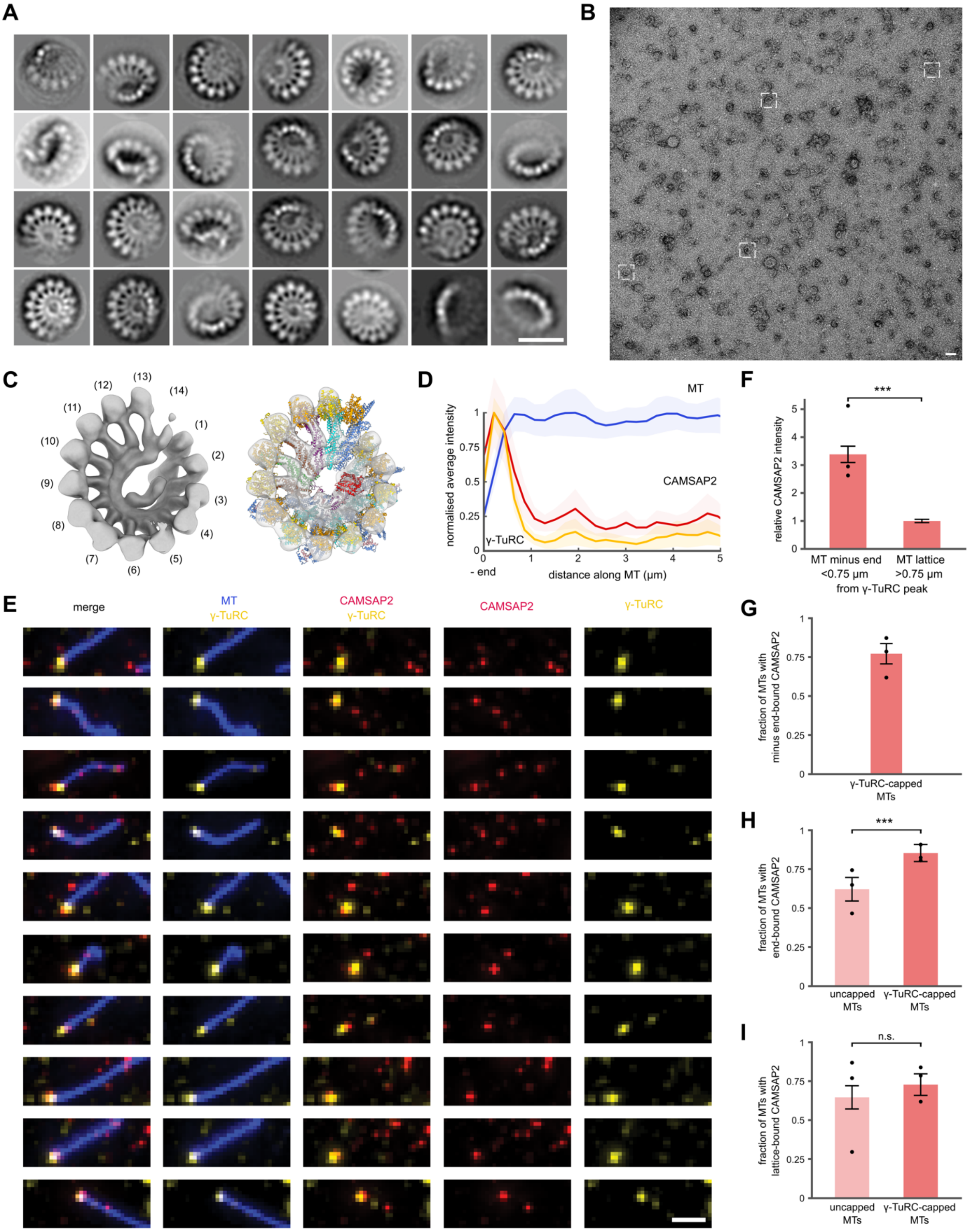

Appendix Figure S8 - Binding of CAMSAP2 to the lattice and γ-TuRC-capped minus ends of MTs nucleated *in vitro*.

A 2D classes obtained from negative stain EM imaging of the recombinant γ-TuRC preparation used for *in vitro* MT nucleation and CAMSAP2 binding experiments. Scale bar represents 25 nm.

B Example negative stain EM micrograph of the recombinant γ-TuRC preparation used for *in vitro* MT nucleation and CAMSAP2 binding experiments. A small number of exemplary γ-TuRC particles are indicated. Scale bar represents 50 nm.

C 3D reconstruction of well-aligning γ-TuRC particles showing 13 stoichiometric and slightly underrepresented spoke 14, likely due to its known conformational plasticity. Subsequent focused classification attempts on spoke 13 and 14 with multiple T-factors revealed no classes indicative of a

population of γ-TuRC with 12 or fewer spokes. Atomic model of the recombinant γ-TuRC (PDB 7QJC) is fit. Actin is displayed in red, MZT1 in pink, otherwise, colouring as in Fig. 2B. Spoke numbers are indicated.
D Normalised average intensity of CAMSAP2 (red), γ-TuRC (yellow) and MT signals (blue) along the length of γ-TuRC-capped MTs observed by multi-colour fluorescence microscopy (n=65). Curves were normalised to peak at 1. Only MTs longer than 5 µm were considered. Data were combined from three biological replicates.
E Gallery of examples illustrating CAMSAP2 (red) colocalising with the γ-TuRC (yellow) and binding to the lattice of *in vitro* nucleated MTs (blue). Scale bar represents 2 µm.
F Average intensity of CAMSAP2 signal within 0.75 µm of the γ-TuRC signal peak (n=795 from 159 MTs, of which 48, 53 and 58 for each respective biological replicate) or further away (n=3068 from 159 MTs). Intensities were normalised to the mean CAMSAP2 intensity on the MT lattice for each biological replicate. $p=4.0x10^{-49}$.
G Fraction of γ-TuRC-capped MTs with CAMSAP2 binding to the minus end (within 1 µm of the MT end; n=159).
H Fraction of γ-TuRC-capped MTs and uncapped MTs with CAMSAP2 binding to either end (within 1 µm of the MT end; n=159 in both conditions, $p=8.63x10^{-7}$).
I Fraction of γ-TuRC-capped MTs and uncapped MTs with CAMSAP2 binding to the MT lattice (i.e., >1 µm from either MT end; n=159 in both conditions, p=0.05806). Dots in (F), (G), (H) and (I) indicate mean values of the three biological replicates; data are shown as mean with 95% confidence interval for all individual data points. *** p<0.001, n.s. non-significant. Significance determined using a one-tailed Welch's t-test.
Source data is available for this figure.

Appendix Table S1 - Mass spectrometry analysis of γ-TuRC-capped MTs isolated from *X. laevis* egg extracts.

α/ β-tubulin, γ-TuRC proteins and known interactors (Bohler *et al*, 2021) are listed according to abundance as ranked by total intensity. Due to genome duplication in *X. laevis*, some proteins are detected as their S and L isoform, corresponding to the long and short chromosomes of homoeologous pairs (Matsuda *et al*, 2015). Source data is available for this table.

Rank	Protein (and isoforms thereof)
1-6, 12, 25	β-tubulin
7-11, 13-20	α-tubulin
21,22,24	actin
71,72	γ-tubulin
181-183	GCP3
185	GCP2
233,305	NEDD1
264,265	GCP4
296-298	CKAP5/XMAP215
307	MZT2B
381	GCP6
408	NME7
483	HAUS5
488	GCP5
636,736	DLGAP5/HURP
662	HAUS1
676	HAUS4
684,685	HAUS6
760	HAUS3
878	XRHAMM/HMMR
926,971	TPX2
951	HAUS8
1285	HAUS2
1410	MZT1

Appendix Table S2 - Cross-correlation of the atomic models of open, partially closed and closed γ-TuRCs indicates that particle supplementation does not bias reconstruction of the MT-capping γ-TuRC, Paclitaxel-stabilised particles only.

We repeated the analysis in Table EV1 for only the set of particles that were stabilised using Paclitaxel, yielding comparable results that underline that particle supplementation does not bias reconstruction of the MT-capping γ-TuRC.

**γ-TuRC conformation** / **Reconstruction**	Open	Partially closed	Closed (hypothetical)
A) Reconstruction of γ-TuRC before supplementation of particles	0.65	**0.68**	0.66
B) Final γ-TuRC-focused reconstruction after supplementation of open particles	0.82	**0.84**	0.79
C) Final γ-TuRC-focused reconstruction after supplementation of simulated closed particles	0.80	**0.84**	0.82
D) Final γ-TuRC-focused reconstruction after supplementation of 50% open particles and 50% simulated closed particles	0.81	**0.85**	0.82
E) Reconstruction of γ-TuRC after supplementation of open particles, followed by local refinement focused on spoke 1-8	0.81	**0.84**	0.79
F) Reconstruction of γ-TuRC after refinement with supplementation of open particles (B), followed by refinement with global sampling without particle supplementation	0.72	**0.75**	0.71
G) Reconstruction of only supplemented open particles	**0.79**	0.76	0.72
H) Reconstruction of only supplemented simulated closed particles	0.76	0.76	**0.81**

Appendix Table S3 - Cross-correlation of the atomic models of open, partially closed and closed γ-TuRCs indicates that particle supplementation does not bias reconstruction of the MT-capping γ-TuRC, DTX-stabilised particles only.

We repeated the analysis in Table EV1 for only the set of particles that were stabilised using DTX, yielding comparable results that underline that particle supplementation does not bias reconstruction of the MT-capping γ-TuRC.

**γ-TuRC conformation**<br>**Reconstruction**	Open	Partially closed	Closed (hypothetical)
A) Reconstruction of γ-TuRC before supplementation of particles	0.73	**0.75**	0.72
B) Final γ-TuRC-focused reconstruction after supplementation of open particles	0.84	**0.86**	0.80
C) Final γ-TuRC-focused reconstruction after supplementation of simulated closed particles	0.81	**0.84**	0.83
D) Final γ-TuRC-focused reconstruction after supplementation of 50% open particles and 50% simulated closed particles	0.82	**0.85**	0.83
E) Reconstruction of γ-TuRC after supplementation of open particles, followed by local refinement focused on spoke 1-8	0.82	**0.84**	0.79
F) Reconstruction of γ-TuRC after refinement with supplementation of open particles (B), followed by refinement with global sampling without particle supplementation	0.75	**0.79**	0.74
G) Reconstruction of only supplemented open particles	**0.81**	0.76	0.70
H) Reconstruction of only supplemented simulated closed particles	0.75	0.75	**0.80**

Appendix Table S4 - Cross-correlation of the atomic models of open, partially closed and closed γ-TuRCs indicates that particle supplementation does not bias reconstruction of the MT-capping γ-TuRC, particles that were not subjected to the shortening procedure only.

We repeated the analysis in Table EV1 for only the set of particles that were not subjected to the shortening procedure during purification, yielding comparable results that underline that particle supplementation does not bias reconstruction of the MT-capping γ-TuRC.

**γ-TuRC conformation**<br>**Reconstruction**	Open	Partially closed	Closed (hypothetical)
A) Reconstruction of γ-TuRC before supplementation of particles	0.70	**0.73**	0.69
B) Final γ-TuRC-focused reconstruction after supplementation of open particles	0.81	**0.84**	0.79
C) Final γ-TuRC-focused reconstruction after supplementation of simulated closed particles	0.78	**0.81**	0.79
D) Final γ-TuRC-focused reconstruction after supplementation of 50% open particles and 50% simulated closed particles	0.81	**0.84**	0.82
E) Reconstruction of γ-TuRC after supplementation of open particles, followed by local refinement focused on spoke 1-8	0.81	**0.83**	0.79
F) Reconstruction of γ-TuRC after refinement with supplementation of open particles (B), followed by refinement with global sampling without particle supplementation	0.75	**0.78**	0.74
G) Reconstruction of only supplemented open particles	**0.77**	0.75	0.71
H) Reconstruction of only supplemented simulated closed particles	0.77	0.77	**0.81**

References

Bohler A, Vermeulen BJA, Wurtz M, Zupa E, Pfeffer S, Schiebel E (2021) The gamma-tubulin ring complex: Deciphering the molecular organization and assembly mechanism of a major vertebrate microtubule nucleator. *Bioessays* 43: e2100114

Goddard TD, Huang CC, Meng EC, Pettersen EF, Couch GS, Morris JH, Ferrin TE (2018) UCSF ChimeraX: Meeting modern challenges in visualization and analysis. *Protein Sci* 27: 14-25

Imasaki T, Kikkawa S, Niwa S, Saijo-Hamano Y, Shigematsu H, Aoyama K, Mitsuoka K, Shimizu T, Aoki M, Sakamoto A *et al* (2022) CAMSAP2 organizes a gamma-tubulin-independent microtubule nucleation centre through phase separation. *Elife* 11

Kollman JM, Greenberg CH, Li S, Moritz M, Zelter A, Fong KK, Fernandez JJ, Sali A, Kilmartin J, Davis TN *et al* (2015) Ring closure activates yeast gammaTuRC for species-specific microtubule nucleation. *Nat Struct Mol Biol* 22: 132-137

Liu P, Zupa E, Neuner A, Bohler A, Loerke J, Flemming D, Ruppert T, Rudack T, Peter C, Spahn C *et al* (2020) Insights into the assembly and activation of the microtubule nucleator gamma-TuRC. *Nature* 578: 467-471

Manka SW, Moores CA (2018) The role of tubulin-tubulin lattice contacts in the mechanism of microtubule dynamic instability. *Nat Struct Mol Biol* 25: 607-615

Matsuda Y, Uno Y, Kondo M, Gilchrist MJ, Zorn AM, Rokhsar DS, Schmid M, Taira M (2015) A New Nomenclature of Xenopus laevis Chromosomes Based on the Phylogenetic Relationship to Silurana/Xenopus tropicalis. *Cytogenet Genome Res* 145: 187-191

Wurtz M, Zupa E, Atorino ES, Neuner A, Bohler A, Rahadian AS, Vermeulen BJA, Tonon G, Eustermann S, Schiebel E *et al* (2022) Modular assembly of the principal microtubule nucleator gamma-TuRC. *Nat Commun* 13: 473
