# Supplementary figures and images for: γ-TuRC asymmetry induces local protofilament mismatch at the RanGTP-stimulated microtubule minus end
Source: EMBO J. 2024 Apr 10;43(10):7. doi: 10.1038/s44318-024-00087-4 (PMC11099078; doi:10.1038/s44318-024-00087-4)

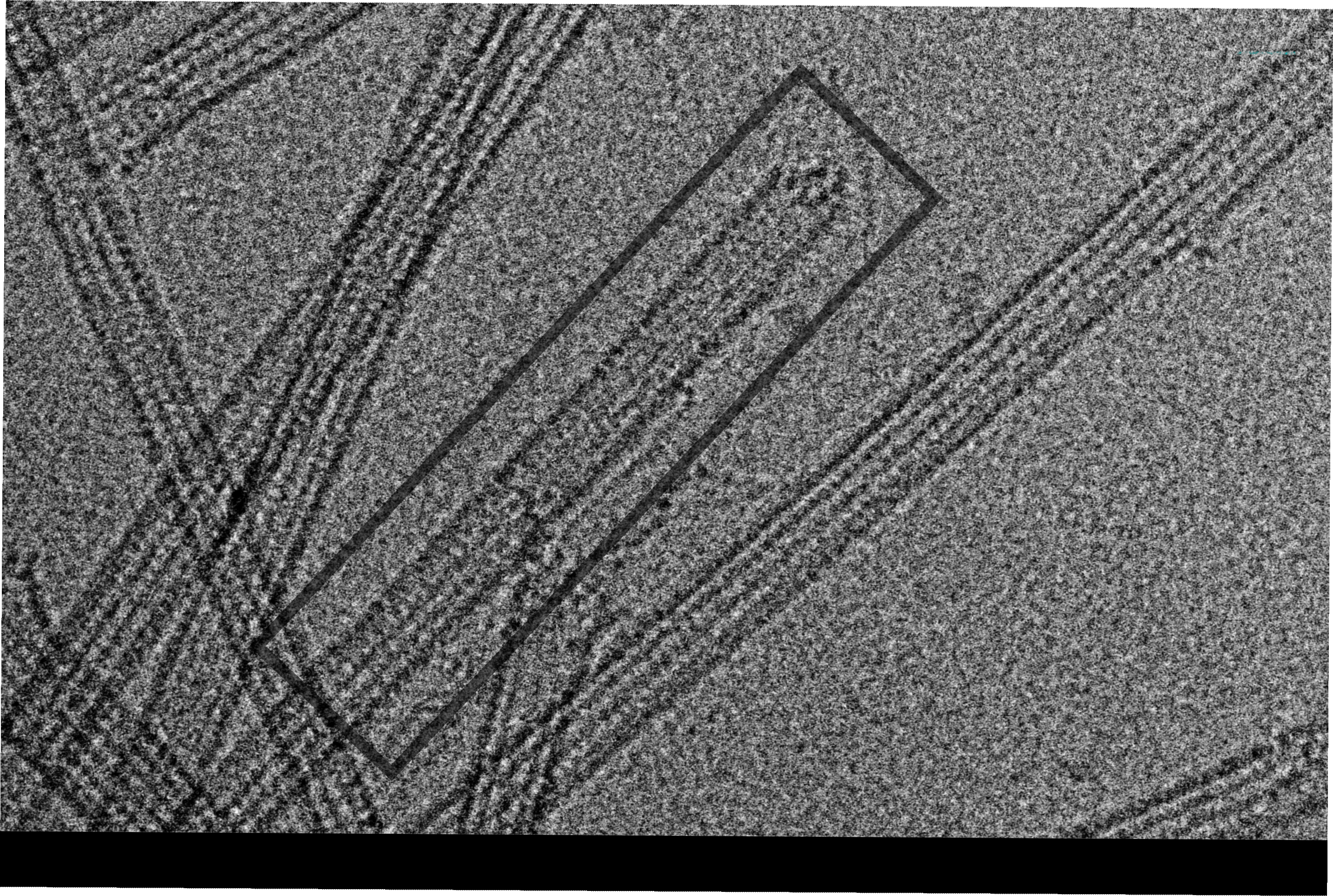

Supplement: Supplementary file 3 — Source data Fig. 1 [file 44318_2024_87_MOESM3_ESM.zip › Figure1ABD/A/SourceData_Figure1A_capped_MT_minus_end2.tiff]

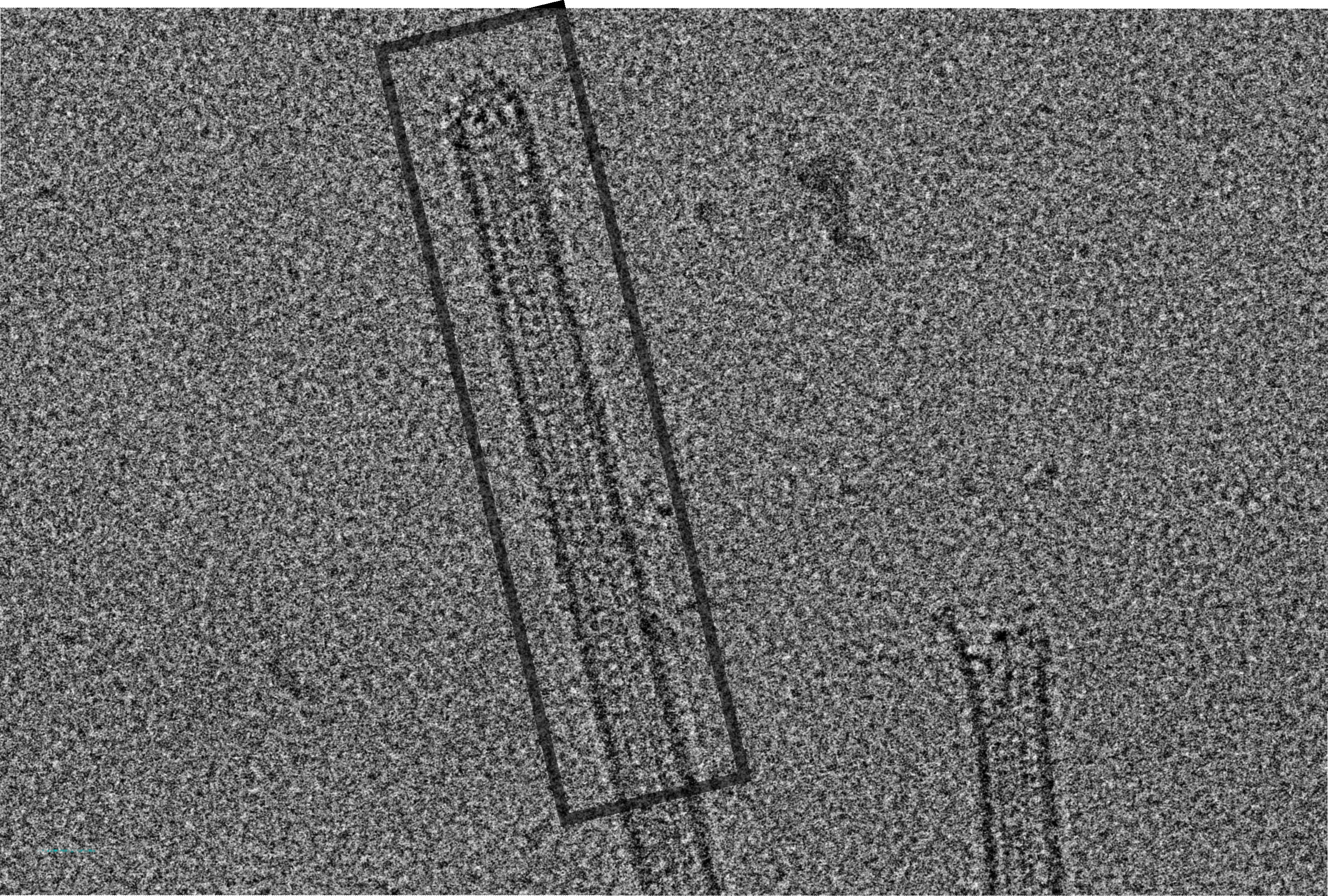

Supplement: Supplementary file 3 — Source data Fig. 1 [file 44318_2024_87_MOESM3_ESM.zip › Figure1ABD/A/SourceData_Figure1A_capped_MT_minus_end1.tiff]

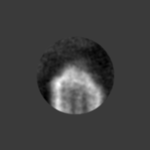

Supplement: Supplementary file 3 — Source data Fig. 1 [file 44318_2024_87_MOESM3_ESM.zip › Figure1ABD/B/SourceData_Figure1B_2Dclass6.png]

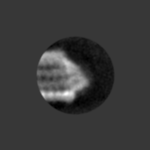

Supplement: Supplementary file 3 — Source data Fig. 1 [file 44318_2024_87_MOESM3_ESM.zip › Figure1ABD/B/SourceData_Figure1B_2Dclass4.png]

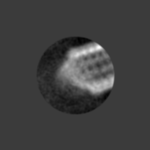

Supplement: Supplementary file 3 — Source data Fig. 1 [file 44318_2024_87_MOESM3_ESM.zip › Figure1ABD/B/SourceData_Figure1B_2Dclass5.png]

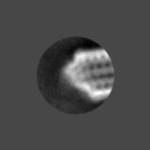

Supplement: Supplementary file 3 — Source data Fig. 1 [file 44318_2024_87_MOESM3_ESM.zip › Figure1ABD/B/SourceData_Figure1B_2Dclass1.png]

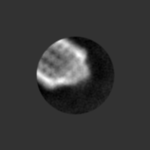

Supplement: Supplementary file 3 — Source data Fig. 1 [file 44318_2024_87_MOESM3_ESM.zip › Figure1ABD/B/SourceData_Figure1B_2Dclass2.png]

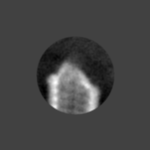

Supplement: Supplementary file 3 — Source data Fig. 1 [file 44318_2024_87_MOESM3_ESM.zip › Figure1ABD/B/SourceData_Figure1B_2Dclass3.png]

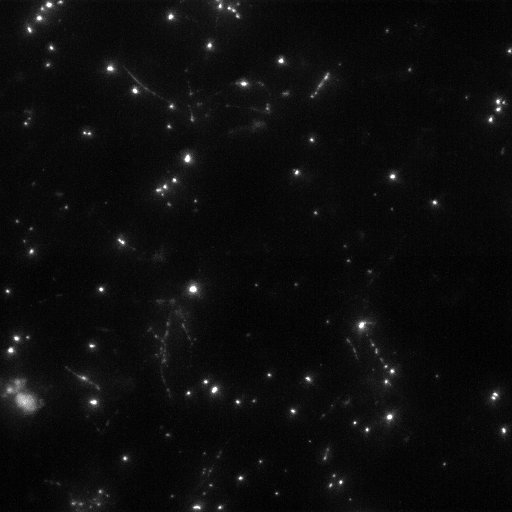

Supplement: Supplementary file 8 — Source data Fig. 4 [file 44318_2024_87_MOESM8_ESM.zip › Figure4/A/SourceData_Figure4A_MT1_ROI_marked.tif]

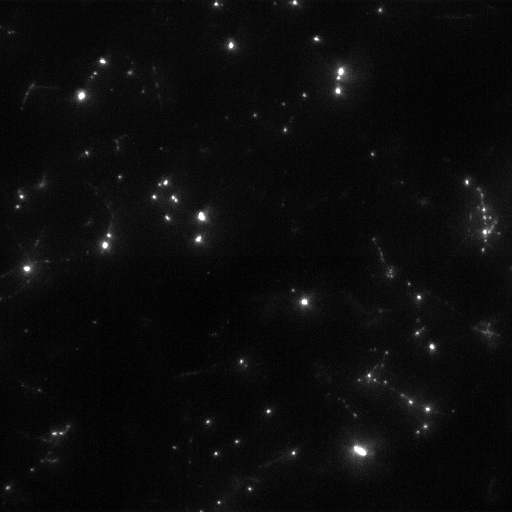

Supplement: Supplementary file 8 — Source data Fig. 4 [file 44318_2024_87_MOESM8_ESM.zip › Figure4/A/SourceData_Figure4A_MT2_ROI_marked.tif]

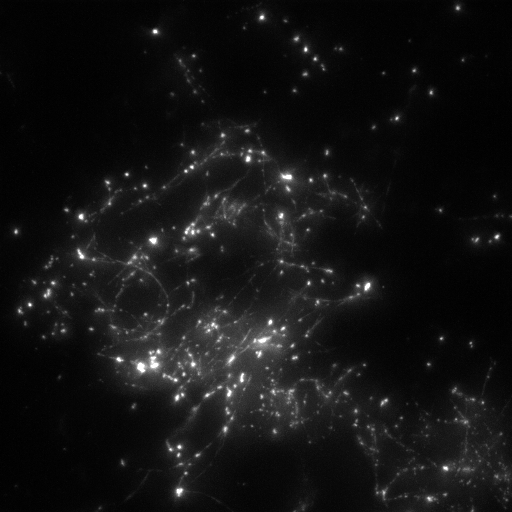

Supplement: Supplementary file 8 — Source data Fig. 4 [file 44318_2024_87_MOESM8_ESM.zip › Figure4/A/SourceData_Figure4A_MT4_ROI_marked.tif]

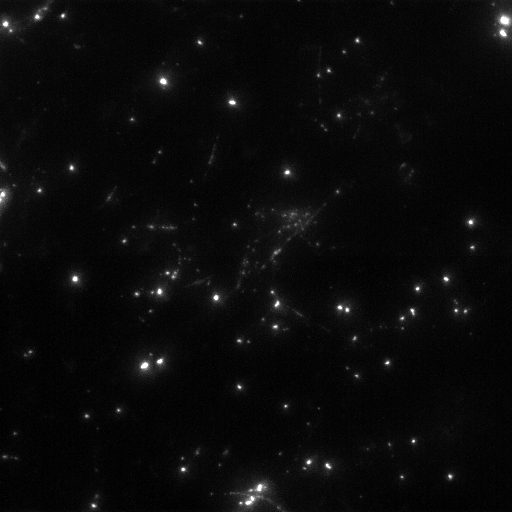

Supplement: Supplementary file 8 — Source data Fig. 4 [file 44318_2024_87_MOESM8_ESM.zip › Figure4/A/SourceData_Figure4A_MT3_ROI_marked.tif]
