# Supplementary material for: γ-TuRC asymmetry induces local protofilament mismatch at the RanGTP-stimulated microtubule minus end
Source: EMBO J. 2024 Apr 10;43(10):7. doi: 10.1038/s44318-024-00087-4 (PMC11099078; doi:10.1038/s44318-024-00087-4)
Supplement: Supplementary file 11 — Expanded View Figures [file 44318_2024_87_MOESM11_ESM.pdf]

Expanded View Figures

Figure EV1. Cryo-EM processing workflow.

(**A**) Detailed image processing scheme. Colours of text and reconstructions indicate the particles used for reconstruction; capped MT minus ends in green, isolated γ-TuRCs in red, a mixture of both in khaki and reference densities in yellow. Respective Fourier Shell Correlation (FSC) curves for resolution values marked by orange and green line are shown in panel (**D**). (**B**) Sample cryo-EM micrograph of γ-TuRC-capped MTs from *Xenopus laevis*, with particles retained for final reconstruction indicated. Scale bar represents 50 nm. (**C**) Comparison of the reconstruction of the γ-TuRC at the MT minus end (orange, left) and the reconstruction of isolated γ-TuRC particles (grey, right) from the same joint refinement run, showing the former reconstruction is not biased by supplementation of isolated γ-TuRC during refinement. Outlined box shows the presence of density for the GRIP2 domain and γ-tubulin of spokes 5 and 6 (indicated by a box) in the γ-TuRC at the MT minus end. This density was subtracted from the supplemented particle images of isolated γ-TuRC and removed from the isolated γ-TuRC reconstruction that served as a reference for refinement. To enable a fair comparison, both reconstructions are low-pass filtered to 17 Å, the resolution of the reconstruction for the γ-TuRC at the MT minus end. Spoke numbering and colouring scheme are indicated. Density for spokes in the background was hidden (clipped) for visual clarity in both panels. (**D**) FSC curve for the γ-TuRC at the MT minus end from γ-TuRC-focused refinement, after removal of supplemented particles (orange) and FSC curve for the MT-dominated local refinement after γ-TuRC-focused refinement (green). Both curves were calculated taking into account a corrected, unbinned pixel size of 2.54 Å/px (see Methods). Threshold at FSC = 0.143 is indicated by a dashed line; the resolution at which the FSC curve first passes the threshold is specified. Source data are available online for this figure.

© The Author(s)

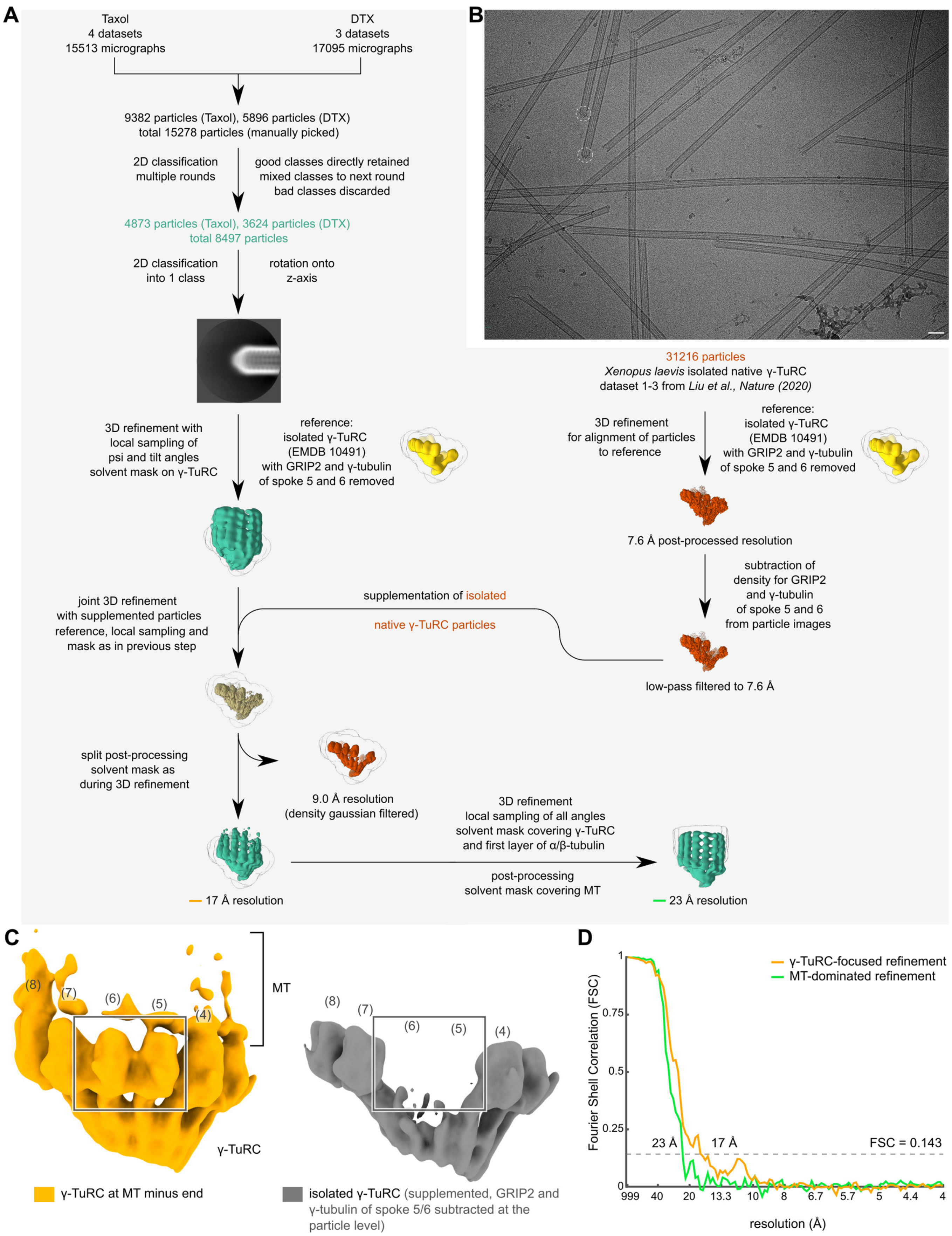
A
Taxol
4 datasets
15513 micrographs
DTX
3 datasets
17095 micrographs
9382 particles (Taxol), 5896 particles (DTX)
total 15278 particles (manually picked)
2D classification
multiple rounds
good classes directly retained
mixed classes to next round
bad classes discarded
4873 particles (Taxol), 3624 particles (DTX)
total 8497 particles
2D classification
into 1 class
rotation onto
z-axis
3D refinement with
local sampling of
psi and tilt angles
solvent mask on γ-TuRC
reference:
isolated γ-TuRC
(EMDB 10491)
with GRIP2 and γ-tubulin
of spoke 5 and 6 removed
B
31216 particles
Xenopus laevis isolated native γ-TuRC
dataset 1-3 from Liu et al., Nature (2020)
3D refinement
for alignment of particles
to reference
reference:
isolated γ-TuRC
(EMDB 10491)
with GRIP2 and γ-tubulin
of spoke 5 and 6 removed
7.6 Å post-processed resolution
subtraction of
density for GRIP2
and γ-tubulin
of spoke 5 and 6
from particle images
joint 3D refinement
with supplemented particles
reference, local sampling and
mask as in previous step
supplementation of isolated
native γ-TuRC particles
low-pass filtered to 7.6 Å
split post-processing
solvent mask as
during 3D refinement
9.0 Å resolution
(density gaussian filtered)
3D refinement
local sampling of all angles
solvent mask covering γ-TuRC
and first layer of α/β-tubulin
post-processing
solvent mask covering MT
17 Å resolution
23 Å resolution
C
(8)
(7)
(6)
(5)
(4)
MT
γ-TuRC
γ-TuRC at MT minus end
isolated γ-TuRC (supplemented, GRIP2 and γ-tubulin of spoke 5/6 subtracted at the particle level)
D
γ-TuRC-focused refinement
MT-dominated refinement
Fourier Shell Correlation (FSC)
1
0.75
0.5
0.25
0
23 Å
17 Å
FSC = 0.143
999
40
20
13.3
10
8
6.7
5.7
5
4.4
4
resolution (Å)

 © The Author(s)

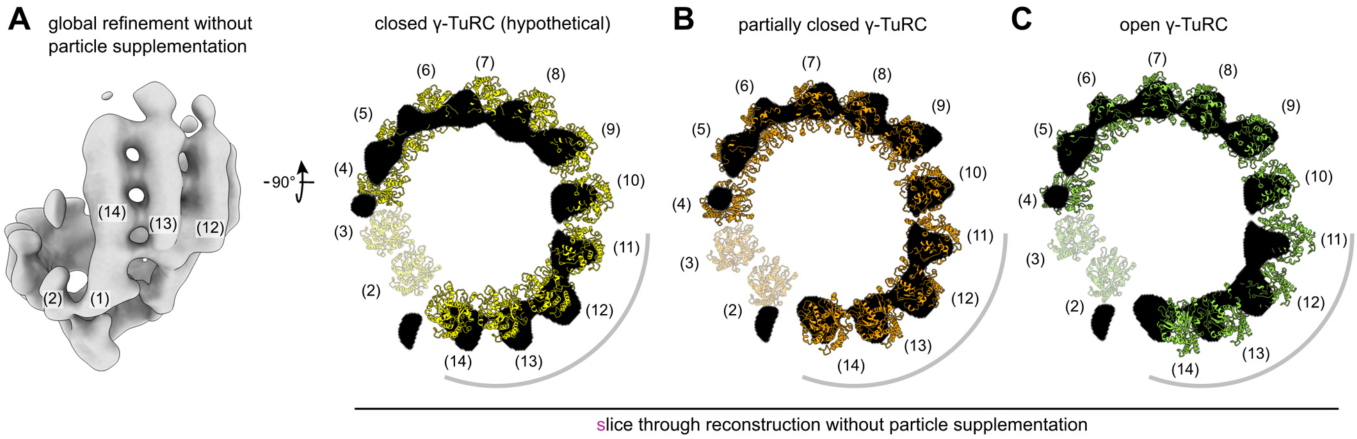

Figure EV2. Refinement and reconstruction of the MT-capping γ-TuRC without particle supplementation confirms a partially closed conformation.

(**A–C**) Refinement of only MT-capping-γ-TuRC particles at global angular sampling, without any particle supplementation, following refinement with particle supplementation. Atomic models for the hypothetical closed γ-TuRC (Kollman et al, 2015) (**A**), the partially closed γ-TuRC (this study, B) and the open γ-TuRC (PDB 6TF9 (Liu et al, 2020), C) were then rigid-body docked based on the GRIP1/2 domains and γ-tubulin molecules of spokes 3 to 9. The partially closed γ-TuRC (**B**) fits the reconstruction well, in spite of the absence of supplemented particles during the refinement, as opposed to the closed (**A**) and open (**C**) γ-TuRC, which clearly deviate from the reconstruction, especially at spokes 11 to 14 (highlighted by a grey line). This shows that the particle supplementation does not induce bias towards the open conformation. For clarity, only a slice through the reconstruction is shown, superposed with γ-tubulin molecules 2–14 of the fit model. The slice was chosen to highlight the fit in the upper half of the spokes. As the slice does not cover spokes 2 and 3, they are shown transparent. Spoke numbering is indicated.

© The Author(s)

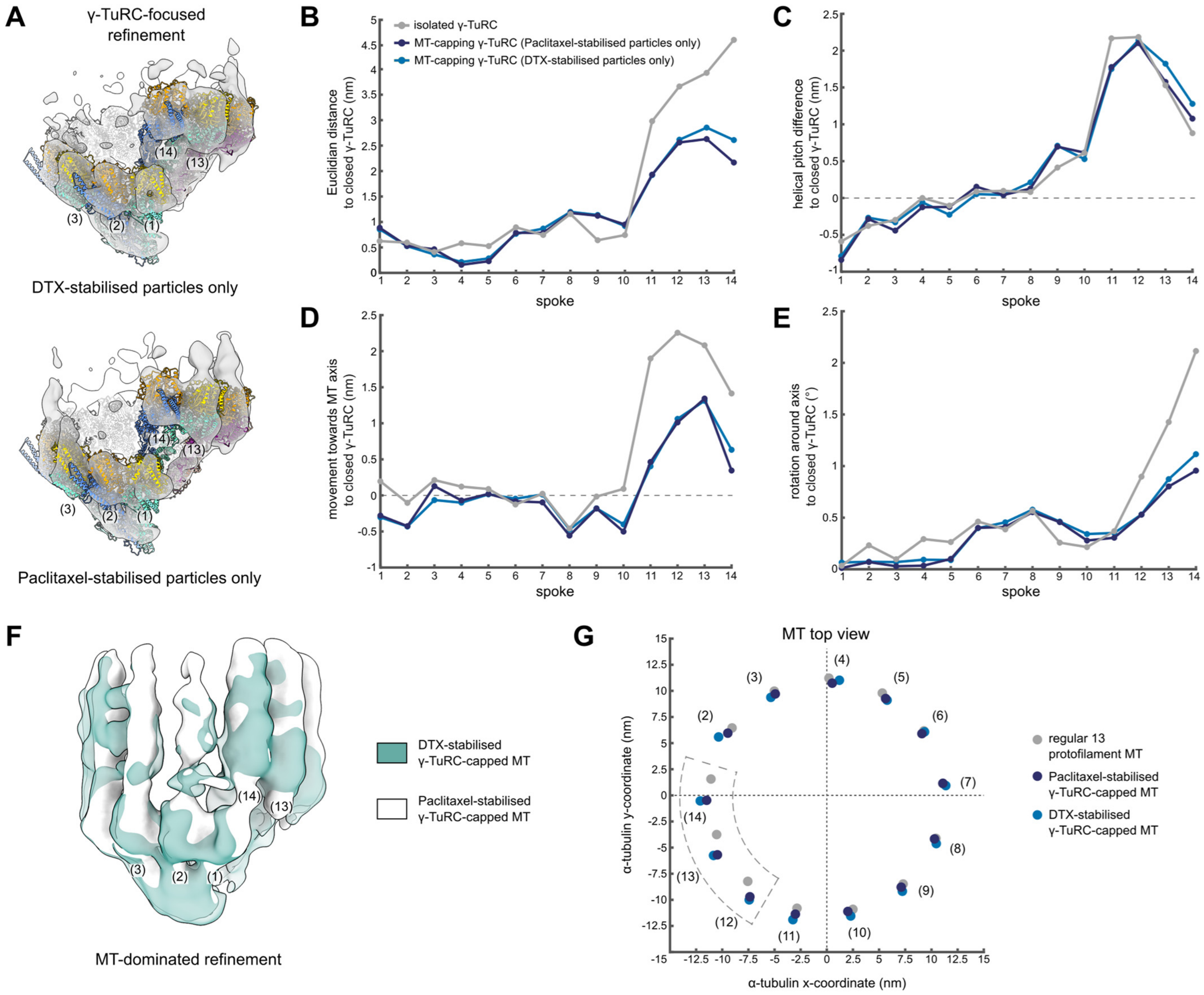

Figure EV3. The conformation of the γ-TuRC and attached MT is not affected by the identity of the MT stabilisation agent.

(**A**) Reconstruction obtained from γ-TuRC-focused refinement of MT-capping γ-TuRCs with only DTX-stabilised particles (top) or only Paclitaxel-stabilised particles (bottom). A model for the γ-TuRC was generated for each reconstruction separately by spoke-wise rigid body docking. A high confidence threshold is used to highlight the fit at the level of γ-tubulin. Spoke numbering is indicated. Colouring as in Fig. 2A. (**B–E**) Euclidian translation distance (**B**), downward change in helical pitch (**C**), translation towards the MT axis (**D**) and rotation around the MT axis (**E**) required to convert the models to the hypothetical, fully closed γ-TuRC for each spoke. Parameters measured from the centre of mass of γ-tubulin. (**F**) Reconstructions of MT-dominated refinements of γ-TuRC-capped MTs with only DTX-stabilised (green) or only Paclitaxel-stabilised particles (white). Density for spokes in the background was hidden for visual clarity. Colouring scheme is indicated. (**G**) Coordinates of the centre of mass for the first α-tubulin in each protofilament in the plane orthogonal to the MT axis for regular 13 protofilament lattice (grey), Paclitaxel-stabilised γ-TuRC-capped MT (dark blue) and DTX-stabilised γ-TuRC-capped MT (light blue). Protofilaments are numbered by the respective spokes in the γ-TuRC. The MT axis is placed on the origin, (0,0). Colouring scheme is indicated. Source data are available online for this figure.

 © The Author(s)

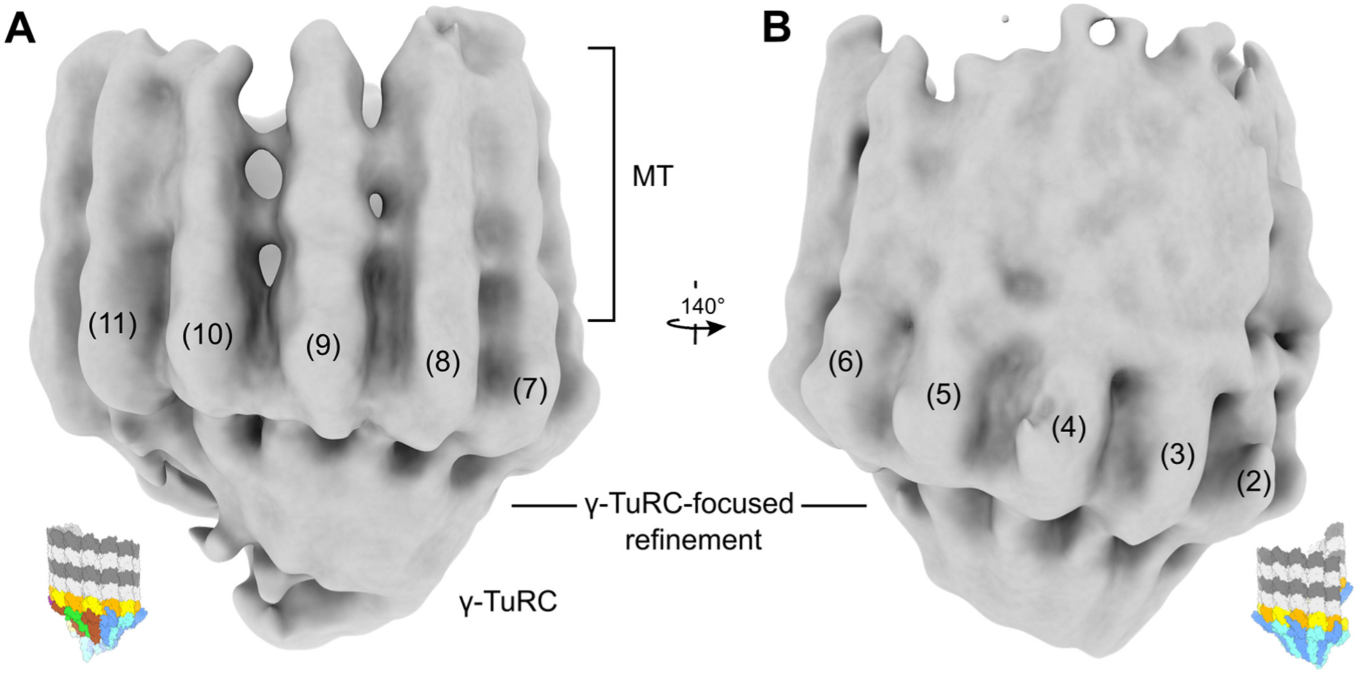

Figure EV4. Not all MT protofilaments are equally rigid with respect to the γ-TuRC.

(**A**, **B**) Refinement focused on the γ-TuRC, in which all γ-TuRC spokes are well-defined, indicating that the γ-TuRC was well-aligned all around. In contrast, the MT density shows local variations in definition: protofilaments associated with spokes 7–11 of the γ-TuRC (**A**) are considerably better defined than those associated with spokes 2–5 (**B**). This indicates increased conformational plasticity of the MT relative to the γ-TuRC at the symmetric side of the γ-TuRC. Reconstructions are local resolution-filtered, shown at low threshold to emphasise MT density. Spoke numbers are indicated. Inset schematics show the orientation of the γ-TuRC.

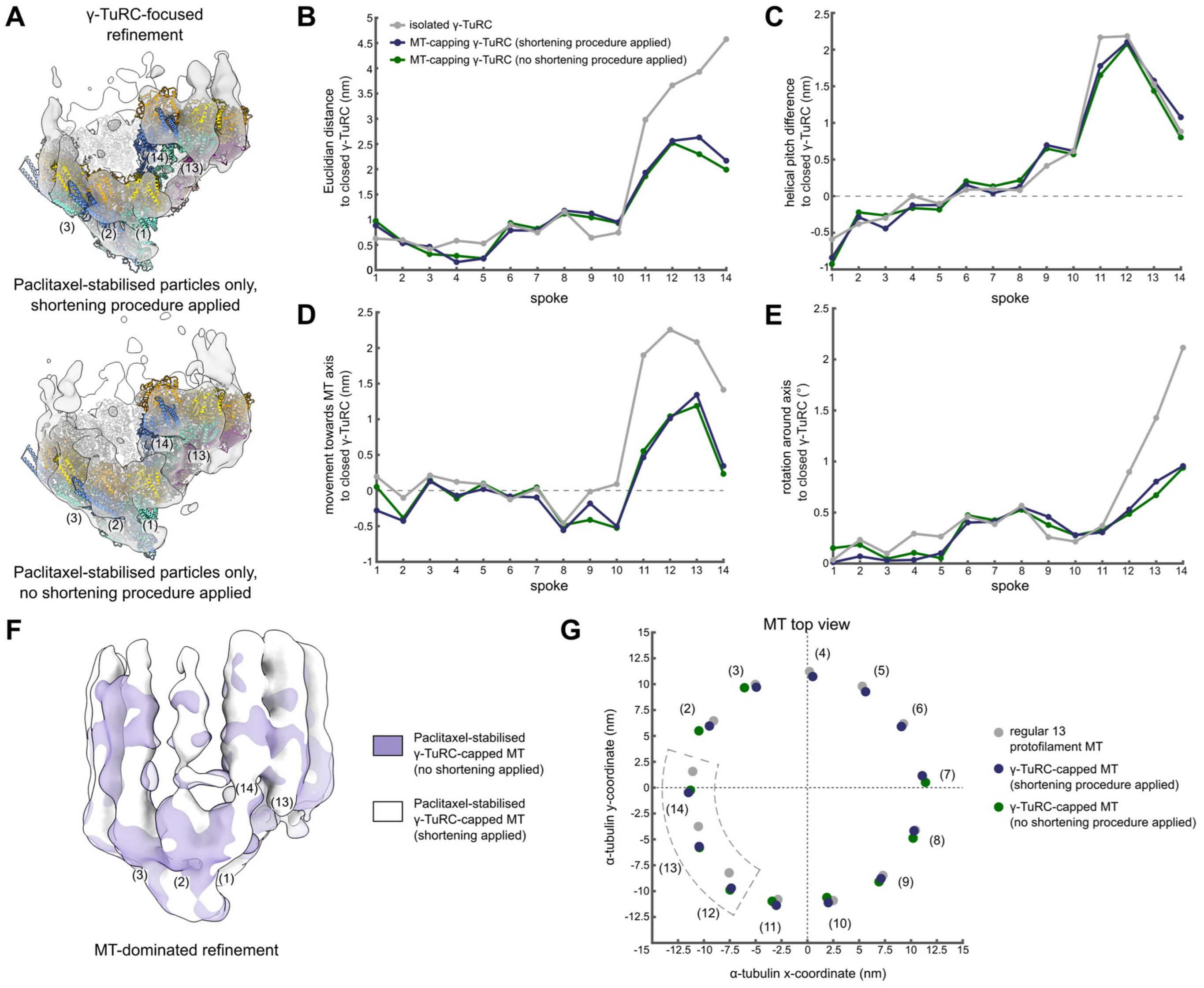

Figure EV5. The conformation of the γ-TuRC and attached MT is not affected by the shortening procedure applied during purification.

(**A**) Reconstruction obtained from γ-TuRC-focused refinement of γ-TuRCs capping Paclitaxel-stabilised microtubules which were (top) or were not (bottom) subjected to the shortening procedure during purification. A model for the γ-TuRC was generated for each reconstruction separately by spoke-wise rigid body docking. A high confidence threshold is used to highlight the fit at the level of γ-tubulin. Spoke numbering is indicated. Colouring as in Fig. 2A. (**B–E**) Euclidian translation distance (**B**), downward change in helical pitch (**C**), translation towards the MT axis (**D**) and rotation around the MT axis (**E**) required to convert the models to the hypothetical, fully closed γ-TuRC for each spoke. Parameters measured from the centre of mass of γ-tubulin. (**F**) Reconstructions obtained from MT-dominated refinements of γ-TuRC-capped Paclitaxel-stabilised MTs which were (white) or were not (purple) subjected to the shortening procedure during purification. Density for spokes in the background was hidden for visual clarity. Colouring scheme is indicated. (**G**) Coordinates of the centre of mass for the first α-tubulin in each protofilament in the plane orthogonal to the MT axis for the regular 13 protofilament lattice (grey), the Paclitaxel-stabilised γ-TuRC-capped MT where shortening was (dark blue) or was not (green) applied during purification. Individual protofilaments could not be fitted at spokes 4 to 6 for the condition without shortening due to lower density quality. Protofilaments are numbered by the respective spokes in the γ-TuRC. The MT axis is placed on the origin, (0,0). Colouring scheme is indicated. Source data are available online for this figure.

 © The Author(s)
